# Toxicological and safety evaluations of *W*e*issella cibaria* strain CMU in animal toxicity and genotoxicity

**DOI:** 10.1007/s43188-021-00119-9

**Published:** 2022-01-04

**Authors:** Laurie C. Dolan, Benjamin G. Arceneaux, Kyung-Hyo Do, Wan-Kyu Lee, Geun-Yeong Park, Mi-Sun Kang, Kyung-Chul Choi

**Affiliations:** 1 GRAS Associates, LLC, 11810 Grand Park Avenue, Suite 500, North Bethesda, MD 20852 USA; 2 Nutrasource Pharmaceutical and Nutraceutical Services, 120 Research Lane, Suite 101, Guelph, ON N1G 0B4 Canada; 3 Laboratory of Veterinary Bacteriology and Infectious Diseases, College of Veterinary Medicine, Chungbuk National University, Cheongju, Chungbuk 28644 Republic of Korea; 4 R&D Center, OraPharm, Inc., 905-ho, 9-16, Yeonmujang-5-gil, Seongdong-gu, Seoul, 04782 Republic of Korea; 5 Laboratory of Biochemistry and Immunology, College of Veterinary Medicine, Chungbuk National University, Cheongju, Chungbuk 28644 Republic of Korea

**Keywords:** *Weissella cibaria*, Safety, Rat, Acute, Subchronic, Mutagenicity, Clastogenicity, NOAEL

## Abstract

*Weissella cibaria* belongs to the *Lactobacillaceae* family and has been isolated from traditional fermented foods and saliva of children with good oral health. Previous investigations have shown that *W. cibaria* CMU (Chonnam Medical University) is expected to be safe based on results of in silico and in vitro analyses. However, there is a lack of studies assessing its safety in vivo. A toxicological safety evaluation of *W. cibaria* CMU was performed using an acute oral safety study in rats, a 14-day oral range finding study, a subsequent 13-week oral toxicity study in rats and a genetic toxicity battery (in vitro bacterial reverse mutation, in vitro chromosome aberration in Chinese Hamster Ovary cells and in vivo micronucleus study in mice). The results of the studies in rats showed that the acute lethal dose of *W. cibaria* CMU is > 5000 mg/kg body weight (bw)/day (1.8 × 10^9^ CFU/kg bw/day) and the 14-day or 13-week no observed adverse effect level (NOAEL) is 5000 mg/kg bw/day (1.8 × 10^9^ CFU/kg bw/day), the highest dose administered. *W. cibaria* CMU was non-mutagenic in the bacterial reverse mutation test and non-clastogenic or aneugenic in vitro and in vivo. In conclusion, the toxicological studies performed demonstrated *W. cibaria* CMU to be a safe strain to consume. This study is the first study examining the potential of a *W. cibaria* strain to cause genetic toxicity and subchronic toxicity in rats according to the Organization for Economic Cooperation and Development guidelines.

## Introduction

The *Weissella* genus includes a number of heterofermentative *Leuconostoc*-like lactic acid bacteria that are generally isolated from fermented foods [1, 2]. *Weissella cibaria* is described as a short, rod-shaped, Gram-positive, non-spore-forming, nonmotile, heterofermentative, and catalase-negative lactic acid bacterium [3]. *W. cibaria* CMU (Chonnam Medical University) has been isolated from saliva samples of children who had little supragingival plaque and no oral diseases including dental caries [4].

Strain CMU has been identified as *W. cibaria* based on whole genome sequence analysis and phylogenetic homology [3–6]. *W. cibaria* is registered as a safe raw material by the Korea Food and Drug Administration (KFDA) and is found in several commercialized oral care probiotics in Korea [7, 8]. *W. cibaria* CMU exhibits several properties for maintenance of oral health, including inhibition of biofilm formation by *Streptococcus mutans*, antibacterial activity against cariogens (*S. mutans* and *Streptococcus sobrinus*) and periodontopathogens (*Fusobacterium nucleatum* and *Porphyromonas gingivalis*) and inhibition of hydrogen sulfide and methyl mercaptan production by *F. nucleatum* and *P. gingivalis* [7].

In studies by Lee et al. [9] and Kang et al. [10], a *W. cibaria* CMU group (0.8 × 10^8^ colony forming units (CFU)/day) was compared with a placebo control group in healthy adults to compare the effects on halitosis and periodontal health and examine tolerability. The results showed no adverse effects of the strain at the level administrated. Available commercialized products provide a daily intake of *W. cibaria* CMU of 1 × 10^9^ to 2.4 × 10^10^ CFU per day. Although *W. cibaria* is on the KFDA Food Ingredient List and is part of the International Dairy Federation List [11], but is not yet listed as a food ingredient for use in the United States or the European Union. This may be because no application has been submitted with this specific goal. It is therefore necessary and essential to conduct a safety assessment for this new strain intended as dietary or food supplements for humans.

The safety of *W. cibaria* CMU has been examined by phenotypic and genotypic analyses designed to assess the potential for antibiotic resistance, antibiotic resistance gene (ARGs) transferability, virulence, hemolysis, mucin degradation, toxic metabolite production, and platelet aggregation [8]. *W. cibaria* CMU strain, both chromosomal and plasmid, was free of ARGs and revealed no ARGs transferability. In addition, the genomic sequence of this strain did not contain any virulence gene, β-hemolysis, mucin degradation, platelet aggregation, and toxic metabolites including d-lactic acid, bile salt deconjugation, ammonia production, β-glucuronidase activity, indole production, nitroreductase activity, phenylalanine degradation, and gelatin liquefaction. The safety of this strain was confirmed by negative reactions. However, animal toxicity or genotoxicity studies on *W. cibaria* strain have not been reported yet.

The purpose of the studies performed for the current evaluation is to add to the current database of safety information on *W. cibaria* CMU, by investigating the potential of the strain to cause toxicity to rats after acute or 14 days or 13 weeks of daily exposure. In addition, genotoxicity assessments including bacterial reverse mutations, chromosome aberrations, and micronuclei formation were also conducted.

## Materials and methods

### Test substance and vehicle

The test substance was *W. cibaria* CMU (oraCMU^®^, OraPharm, Inc., Seoul, Korea) in a vehicle of distilled water (Daihan Pharma Co., Seoul, Korea). The *W. cibaria* preparation contained 3.6 × 10^8^ CFU/g. Preparations used for each study were prepared daily. For the in vivo studies, samples were collected from the dosage formulations to verify homogeneity and dose concentration on the day of administration.

Cell viability was determined using an EZ-Cytox cell viability assay kit (iTSBiO, Korea). Briefly, EZ-Cytox kit reagent was added into each well for 2 h under standard culture conditions, after which 96-well plates were gently shaken thoroughly for 5 min on a rocker at room temperature. The absorbance of the treated and untreated samples at 450 nm was then measured on a multi-well microplate reader (VersaMax, Molecular devices, San Jose, CA, USA). DMEM medium supplemented with the same volume of kit reagent on an empty well was used as a blank. Cell viability was represented by percentage values compared to a control and then used to calculate the number of viable cells.

### Study conduct

All studies conducted in animals complied with the guidelines of animal ethics and were approved by the IACUC of Korea Conformity Laboratories (KCL). Approval numbers for each study are as follows: In vivo micronucleus test (IA20-02198); single oral dose toxicity study (IA20-01118); thirteen-week repeated oral Dose toxicity study (IA20-01554). All studies were performed at KCL in Incheon, Korea.

The 14-day dose range finding and 13-week studies were performed according to OECD guidelines 407 and 408, respectively [12, 13]. OECD guidelines 417, 473 and 474 were followed for the bacterial reverse mutation study, the in vitro chromosomal aberration test, and the in vivo micronucleus test, respectively [14–16]. All studies were conducted in compliance with non-clinical trial management standards as stated in the Korean Ministry of Food and Drug Safety (MFDS) Notification No. 2018-93, “Principles of Good Laboratory Practices (GLP)” (November 21, 2018), except for the 14-day oral dose range finding study. All of the studies were also performed according to MFDS Notification No. 2017-71, “Toxicity Test Standards of Medicine and Medicinal Supplies” (August 30, 2017).

### Animals

Specific Pathogen Free (SPF) Sprague–Dawley (SD) rats from Orient Bio Inc. (Seongnam, Korea) were used in this study. Animals were acclimated for 5–8 days (depending on the study) before treatment and were approximately 8 weeks of age at the start of the acute study, and 6 weeks for the 14-day and 13-week studies. Animals were housed in wire mesh cages in groups no larger than three for the acute and 14-day studies and five for the 13-week study. The animal housing room was maintained on a 12-h light/dark cycle, with temperature and relative humidity of 24 ± 0.3 °C and 46.3 ± 1.5% in the acute study, 23.3 ± 0.5 °C and 51.1 ± 0.5% in the 14-day study and 22.7 ± 0.4 °C and 54.9 ± 1.5% in the 13-week study. Animals were supplied diet (Teklad Certified Irradiated Global 18% Protein Rodent Diet, Envigo, USA) and filtered water ad libitum. Animals were selected for use in the study only if their body weight fell within 20% of the mean. For the acute study, animals were fasted overnight prior to dosing and food was returned three to four hours after dosing; water was provided ad libitum during fasting. For the 14-day and 13-week studies, rats were randomly allocated into treatment groups to ensure adequate distribution of body weights.

### Single dose oral toxicity study

Animals were divided into four groups (n = 5/sex/group), one of which was a vehicle control group (distilled water), and three test groups receiving the test material at 1250, 2500, or 5000 mg/kg body weight (bw). The viable cell counts in the low, medium, and high dose groups were 4.5 × 10^8^, 9.0 × 10^8^, and 1.8 × 10^9^ CFU/kg, respectively. The test material was prepared just prior to dosing by dissolving the test material in distilled water at the appropriate concentration. Animals were dosed by gavage at a volume of 15 mL/kg bw. Following dosing, general clinical signs in all animals were observed more than once a day for 14 days before necropsy. On the day of administration, clinical signs were observed in all animals at 30 min and every hour until 6 h after administration. Body weights were taken at acquisition, grouping, immediately prior to dosing, and on days 1, 4, 7, and 14 after dosing. On day 14, animals were anesthetized with carbon dioxide (CO_2_) and humanely euthanized. Complete postmortem examinations were performed on all organs.

### 14-day dose range finding study

In total, 40 rats (n = 20/sex) were assigned to four treatment groups (n = 5/sex/group)—vehicle control (Group 1) or the following amounts of the test substance: 4.5 × 10^8^ CFU/kg bw/day (Group 2), 9.0 × 10^8^ CFU/kg bw/day (Group 3) or 1.8 × 10^9^ CFU/kg bw/day (Group 4) for 14 days. Individual doses were calculated based on the most recent weekly body weights and were adjusted each week to maintain the targeted dose level for all rats (i.e., mg/kg bw/day). All doses were administered volumetrically at 15 mL/kg by oral gavage.

General clinical signs of all animals were observed and recorded daily. Body weights were recorded at acquisition, grouping, before administration, once a week during the study, and before necropsy. Food intake was measured right before the first administration and once a week during the study. Food consumption per animal was measured by the average intake (g/rat/day) of individual animals in a cage. Water consumption was measured in a similar manner to food consumption. The night before termination, the animals were subjected to an overnight fast in which water was still provided ad libitum. At study termination, animals were euthanized under isoflurane anesthesia and blood samples were drawn from abdominal aorta. Whole blood collected on EDTA-K2 was used for hematological analyses (white blood cell count (WBC), neutrophils (NEU), eosinophils (EOS), basophils (BAS), lymphocytes (LYM), monocytes (MON), large unstained cells (LUC), percent of neutrophils (NEP), percent of eosinophils (EOP), percent of basophils (BAP), percent of lymphocytes (LYP), percent of monocytes (MOP), percent of large unstained cells (LUP), red blood cell count (RBC), hemoglobin concentration (HGB), red cell distribution width (RDW), hematocrit (HCT), mean corpuscular volume (MCV), mean corpuscular hemoglobin (MCH), mean corpuscular hemoglobin concentration (MCHC), reticulocytes (RET), platelets (PLT), and mean platelet volume (MPV)). Serum was obtained from whole blood collected on 3.2% sodium citrate and analyzed for clinical chemistry (aspartate aminotransferase (AST), alanine aminotransferase (ALT), gamma-glutamyl transferase (GGT), alkaline phosphatase (ALP), total bilirubin (BIL), blood urea nitrogen (BUN), creatinine (CRE), uric acid (UA), glucose (GLU), total cholesterol (CHO), triglycerides (TG), total protein (PRO), albumin (ALB), ALB/GLOB ratio, lactate dehydrogenase (LDH), creatine phosphokinase (CPK), calcium (Ca), inorganic phosphorus (IP), magnesium (Mg), sodium (Na), potassium (K), and chloride (Cl)).

At necropsy, postmortem examinations were completed on all organs. Weights of the following organs were obtained: testes, prostate, ovaries, uterus, spleen, liver, thymus, adrenals, kidneys, heart, lungs, brain, and pituitary gland. All collected organs were preserved in 10% phosphate-buffered formalin except for testes, which were fixed in Böuin solution. Histopathological examination was not performed.

### 13-week repeated dose toxicity study

Eighty animals (n = 40/sex) were assigned to four treatment groups (n = 10/sex/group)—vehicle control (Group 1), and three treatment groups receiving the following amounts of test substance: 4.5 × 10^8^ CFU/kg bw/day (Group 2), 9.0 × 10^8^ CFU/kg bw/day (Group 3) or 1.8 × 10^9^ CFU/kg bw/day (Group 4). Dose levels were adjusted based on body weights measured right before each administration, dosing volume was 15 mL/kg bw/day, and each dose was given by oral gavage for 13 weeks.

General clinical signs were observed and recorded daily for all animals. Individual body weights were recorded at acquisition, grouping, right before administration, once a week during exposure, and right before necropsy. Food consumption was measured right before first administration and once a week during the rest of the study. Food intake per day and food intake per animal were calculated the same way as for the 14-day dose range finding study. Ophthalmic examinations were conducted on all animals before first administration and during the last week of the study. During the last week of test substance administration, urine was collected from five animals per sex per group using metabolic cages. Urine samples were analyzed for the following parameters: color, glucose, bilirubin, ketone bodies, specific gravity, occult blood, pH, protein, urobilinogen, nitrite, leukocytes, erythrocytes, epithelial cells, and casts. Animals were fasted overnight before termination, but water was provided ad libitum*.* On the day of termination, all animals were anesthetized with isoflurane and blood samples were drawn from the abdominal aorta. Blood samples collected on EDTA-K2 were analyzed for the same hematology parameters as described for the 14-day dose range finding study, but methemoglobin (metHGB) was added. Analyses for coagulation (prothrombin time (PT) and active partial thromboplastin time (APTT)) were added. Serum clinical chemistry was also the same as the 14-day study, with the addition of bile acid (BA), high density lipoprotein (HDL), low density lipoprotein (LDL), cholinesterase (ChE), thyroxine (T4), triiodothyronine (T3), and thyroid stimulating hormone (TSH).

Scheduled necropsy was completed on all surviving animals and complete postmortem examinations were performed on all organs. Vaginal smear examinations were completed in all female rats prior to necropsy to determine estrus cycle by the shape of epithelial cells using Giemsa staining (Sigma, St. Louis, MO, USA). Weights of the 13 organs weighed in the 14-day study, plus the epididymides and thyroid, were taken at necropsy. Both sides of bilateral organs were weighed and preserved, and the thyroid was fixed prior to weighing. Organs and tissues from all animals were preserved in 10% neutral phosphate-buffered formalin (other than the testes and epididymides, which were fixed in Böuin solution, and the eyes which were fixed in Davison’s solution). Preserved organs included the brain, pituitary, heart, lungs, liver, kidney, urinary bladder, mesenteric lymph node, thymus, spleen, adrenal gland, esophagus, aorta, spinal cord, sciatic nerve, skeletal muscle, skin, mammary gland, eye, stomach, colon, rectum, femur, sternum and bone marrow, trachea, tongue, prostate gland, testes, epididymis, seminal vesicle, pancreas, salivary gland, submandibular lymph node, thyroid and parathyroid glands, duodenum, jejunum, ileum, cecum, ovary, uterus, cervix, and vagina. Specimens from the vehicle control and high dose group were prepared and examined for all fixed organs and tissues.

### Bacterial reverse mutation assay

Bacterial strains utilized in all experiments were *Salmonella typhimurium* tester strains TA98, TA100, TA1535 and TA1537 and *Escherichia coli* WP2*uvrA*. Experiments were conducted in the absence and presence of an S9 metabolizing system, using the preincubation method. The test product was formulated as a solution in distilled water to provide dose levels of up to 5000 μg/plate. The positive control chemicals sodium azide (NaN_3_), 9-aminoacridine (9-AA), 2-(2-furyl)-3-(5-nitro-2-furyl) acrylamide (AF-2), 2-aminoanthracene (2-AA), benzo(a)pyrene (BP), and 4-nitroquinoline 1-oxide (4-NQO) and their vehicle dimethyl sulfoxide (DMSO) were sourced from Sigma, with the exception of AF-2 and DMSO, which came from Wako (Tokyo, Japan) and Junsei (Tokyo, Japan) (respectively). The bacterial strains and the S9 tissue fraction (which was isolated from livers of SD rats induced by intraperitoneal injection of Aroclor-1254) were sourced from Molecular Toxicology, Inc (Boone, NC, USA).

For the experiments, the following substances were mixed in a test tube and poured over the surface of a minimal glucose agar plate (Molecular Toxicology, Inc.): (1) the positive or negative control solutions or test formulations (0.05 mL each); (2) 0.5 mL of 0.1 M phosphate buffered saline or 0.5 mL of the S9 mix (for metabolic activation); (3) 0.1 mL of bacterial suspension; and (4) 2.0 mL of overlay agar supplemented with biotin and limited amounts of histidine and tryptophan (Molecular Toxicology, Inc.). Concentrations of test article used were 62, 185, 556, 1667, and 5000 μg/plate. The positive controls in the absence of S9 mix were AF-2 for *S. typhimurium* TA98 and TA100, sodium azide for TA1535, 9-AA for TA1537 and 4-NQO for *E. coli* WP2*uvrA*. The positive control for all bacterial strains in the presence of S9 mix was 2-AA, except for TA98, which used BP. Distilled water served as the negative control. Three replicate plates were prepared for each test condition. After exposure for 20 min at 37 °C, and incubation for 48 h at 37 °C, the number of colonies per plate was counted manually with an electronic register (Model 570, SUNTEX, Taipei, Taiwan). The test result was recorded as experimental value, average value, and standard deviation for the number of revertant colonies per plate. The result was deemed “positive” if there was a dose-dependent increase and/or a reproducible increase at one or more concentrations in the number of revertant colonies per plate in at least one strain with or without metabolic activation.

### Chromosomal aberration assay

Chinese Hamster Ovary cells (CHO-k1) were obtained from the Korean Cell Line Bank (KCLB, Seoul, Korea). The cells were cultured in F12 nutrient mixture (GIBCO, Grand Island, NY, USA) and 10% fetal bovine serum (Corning, NY, USA) at 37 °C and 5% CO_2_ and were subcultured every 3–4 days. The S9 preparation used for metabolic activation is described under the methods for the bacterial reverse mutation assay.

A preliminary dose range finding study was performed with eight concentrations up to 5000 µg/mL to determine the highest concentration used for each exposure scenario (24 h incubation without S9 and 6 h treatment and 18 h recovery with or without S9). For the 24-h incubation scenario, the relative increase in cell counts (RICC) was 46.40% and 60.39% in for 185.19 µg/mL and 61.73 µg/mL, respectively. For the 6 h exposure group without S9 mix, RICC were 46.77% and 61.55% for 555.56 µg/mL and 185.19 µg/mL, respectively. For the 6 h exposure group with S9 mix, RICC were 49.64% and 61.37% for 1666.67 µg/mL and 555.56 µg/mL, respectively. Based on these results, the maximum concentrations chosen for use in the chromosome aberration study were 185.19 µg/mL for 24 h, 555.56 µg/mL for 6 h-S9 and 1666.67 µg/mL for 6 h + S9. Two lower concentrations were also used in each assay, at threefold dilutions from the highest concentrations.

For the assay, the positive control substance was mitomycin C without metabolic activation (S9 mix), and cyclophosphamide with S9 mix. The negative control was distilled water. Approximately 22 h after treatment, colcemid (GIBCO) was added to each culture plate for a final concentration of 0.2 µg/mL. The cultures were incubated for an additional 2 h, after which they were detached using 1X trypsin solution. The medium containing mitotic cells was centrifuged at 1000 rpm for 5 min. The cell pellets were resuspended in 75 mM potassium chloride solution and incubated at 37 °C for 20 min. The cells were fixed 3 times with Carnoy’s fixative solution (acetic acid:methanol = 1:3 v/v) for slide preparation. The slides were stained with 5% Giemsa solution for 5 min and observed microscopically. Two slide samples were prepared from each plate and 150 metaphase cells per plate were counted.

Structural (gaps, breakage, exchange) and numerical aberrations were evaluated. Aneuploidy was not counted as numerical aberration since it occurs frequently in cell-lines such as CHO-k1. Endoreduplication was classified as polyploidy, and it was recorded if endoreduplication was frequently observed. The test material was considered to be clearly positive for clastogenicity (and/or aneugenicity) if at least one of the test concentrations exhibited a statistically significant increase compared with the corresponding negative control, the increase was dose-related and any of the results were outside of the distribution of historical negative control data.

### Micronucleus assay

The study was conducted to evaluate the potential genotoxicity of the test material in an in vivo micronucleus assay. Male SPF CrlOri:CD1 (ICR) mice (n = 25) from Orient Bio Co., Ltd were used and divided into five equal groups: one negative control, one positive control and three treatment groups (1250, 2500 and 5000 mg/kg bw/day). The positive control used in the study was mitomycin C (2.0 mg/kg bw/day) and the negative/vehicle control was distilled water. Animals were dosed with the test material orally once a day for two days at 24-h intervals after being subjected to a 3–4 h fast, Mitomycin C was administered intraperitoneally rather than orally. Animals were observed on the day of administration from 30 min to 4 h after administration and at the time of autopsy for the occurrence of dead animals and abnormal signs. All animals were weighed before acquisition and grouping, administration of test substances, and before bone marrow collection. At 18 and 24 h after the last dose, all animals were euthanized, and bone marrow was collected from the thigh bone and suspended in fetal bovine serum. Bone marrow smears (3/animal) were prepared on glass slides at room temperature and fixed in methanol for 5 min. To score the polychromatic erythrocytes (PCE) to normochromatic erythrocytes (NCE) ratio, slides were stained with 4% Giemsa solution. To score the micronucleated polychromatic erythrocytes (MNPCE) from PCEs, the slides were fixed, stained with acridine orange, and covered with cover glasses.

The observation of slides was performed with the investigator blinded to test condition. The PCE to NCE ratio was determined by optical microscope at greater than 1000 × magnification. MNPCEs were observed by fluorescence microscope equipped with an FITC filter and more than 400 × magnification. The PCE/NCE ratio was determined by scoring the number of PCEs and NCEs in 500 erythrocytes per animal. Micronucleus frequency was determined by analyzing the number MNPCEs from 4000 PCEs per animal. Under the fluorescence microscope, PCEs appeared as a red-fluorescent and did not stain for nuclei using acridine orange. NCEs appeared only as shadow entities without fluorescence. With the optical microscope, PCEs stained with Giemsa appeared blue or purple, and NCEs appeared pink. In order to judge the presence of micronuclei, the largest size was defined as ½ the size of erythrocyte diameter and the smallest size was the limit of identification. Shapes included circle, donut, and semi-circle and color was defined as the same as the nucleus of the cell.

The result was considered positive if all of the following acceptability criteria were met: (1) the frequency of MNPCE/4000 PCEs (mean ± SD, %) is statistically reliable, increases dose-dependently, and/or shows a reproducible positive reaction at one or more concentrations, and (2) any of the results are outside the distribution of historical negative control data.

### Statistical analysis

The statistical package SPSS 12.0 K (SPSS, Chicago, IL, USA) was used for all analyses. The criterion for significance was *p* < 0.05. In the single dose oral toxicity study, body weight data were analyzed using the one-way analysis of variance (ANOVA) test to detect any differences between groups. In the 14-day dose range finding study, differences between groups were examined by the one-way ANOVA test. If a significant difference was detected, the Duncan test was used for data comparison if there was homogeneity of variance, and Dunnett’s *T* test was used if there was not homogeneity of variance. In the 13-week toxicity study, continuous data were compared using the one-way ANOVA test to determine if there was a difference between treated animals and controls. If there was a significant difference, the data were analyzed similarly to the 14-day study. Detailed clinical signs, urinalysis, urine sediments, and estrus cycle data were analyzed as non-continuous data and were converted by scale conversion and analyzed by the Chi-squared test. In the chromosome aberration assay, the number of aberrant metaphases, excluding gaps, and the incidence of polyploid plus endoreduplication were analyzed. Comparisons between groups were performed using the Chi-squared test. A linear logistic regression test was used to determine if the response was dose dependent. In the micronucleus assay, the negative control and treatment groups were compared by one-way ANOVA. If a significant difference was detected, the data were analyzed similarly to the 14-day study.

## Results

### Single dose oral toxicity with *W. cibaria* CMU

There were no mortalities, abnormal clinical signs, or macroscopic findings in the study. Normal weight gain was observed and there were no significant body weight changes between the test groups and the vehicle control group. Under the conditions of the study, the lethal dose was greater than 5000 mg/kg bw (1.8 × 10^9^ CFU/kg bw) (data not shown).

### 14-day dose range finding study with *W. cibaria* CMU

There were no mortalities, abnormal clinical signs, or macroscopic findings in the study. There were no significant changes in body weight, body weight gain, food or water consumption, or organ weights between the test groups and the vehicle control group. Findings that were significantly different in treated compared to control animals are shown in Table 1. The MPV of males receiving 2500 and 5000 mg/kg bw/day, NEP in females receiving 1250 and 2500 mg/kg bw/day, and BUN and TG in females receiving 1250 mg/kg bw/day were increased compared to the vehicle control group. The changes that were observed were not considered to be toxicologically relevant by the study investigators due to lack of dose dependency and/or minor nature of the changes. The highest dose to be used in the 13-week study was chosen as 5000 mg/kg bw/day (1.8 × 10^9^ CFU/kg bw/day) based on the results.

**Table 1:** Parameters that exhibited changes in the 14-day study with *W. cibaria* CMU

Parameter	Control	1250 mg/kg bw/day	2500 mg/kg bw/day	5000 mg/kg bw/day
Males				
MPV (fL)	6.1 ± 0.4	6.1 ± 0.2	6.6 ± 0.2^*^	6.6 ± 0.2^*^
Females				
NEP (%)	7.8 ± 3.4	15.0 ± 4.4^*^	15.2 ± 4.9^*^	11.3 ± 4.2
BUN (mg/dL)	14.3 ± 1.9	17.2 ± 1.2^*^	13.1 ± 2.1	15.0 ± 2.3
TG (mg/dL)	20 ± 10	33 ± 10^*^	20 ± 5	19 ± 3

*BUN* blood urea nitrogen, *dL* deciliter, *fL* femtoliters, *MPV* mean platelet volume, *NEP* percent of neutrophils, *TG* triglycerides

N = 5/sex/group. Data are presented as mean ± standard deviation

^*^Significant difference compared with the control group value, *p* < 0.05

### 13-week repeated dose toxicity study with *W. cibaria* CMU

**Fig. 1:**
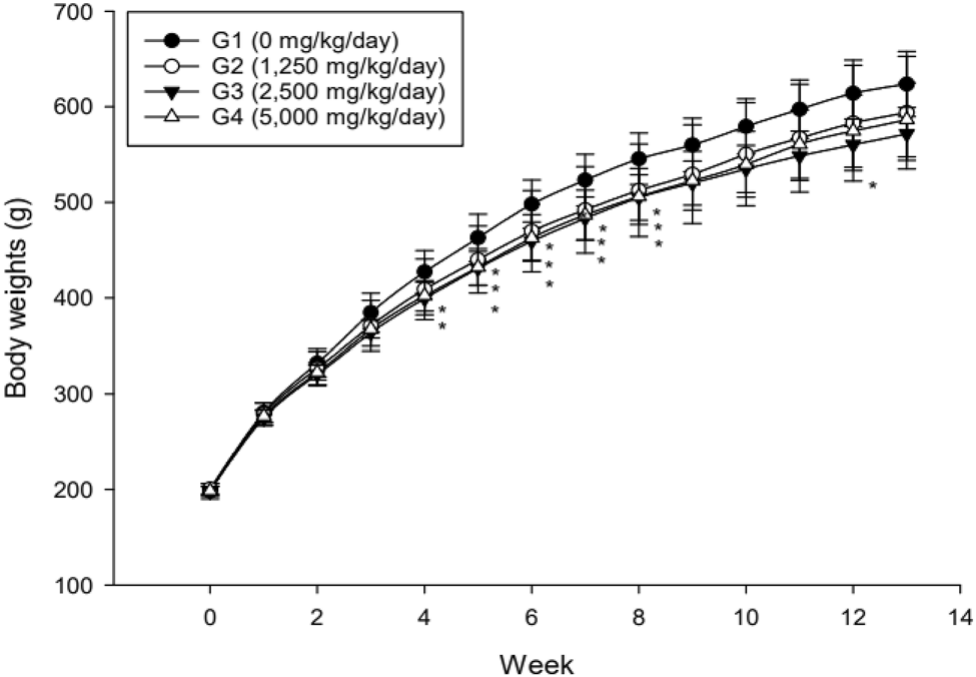
Body weights of male rats in 13-week study of *W. cibaria* CMU. Individual animal weight was recorded at acquisition, grouping, at the before administration, once a week during the study and before necropsy. Body weights of males in the 2500 and 5000 mg/kg bw/day groups were less than controls at week 4, all groups of treated males exhibited lower body weights than controls from weeks 5–8, and males in the 2500 mg/kg bw/day also had lower body weights than controls at week 12. ^*^Significant difference compared with control group, *p* < 0.05

**Fig. 2:**
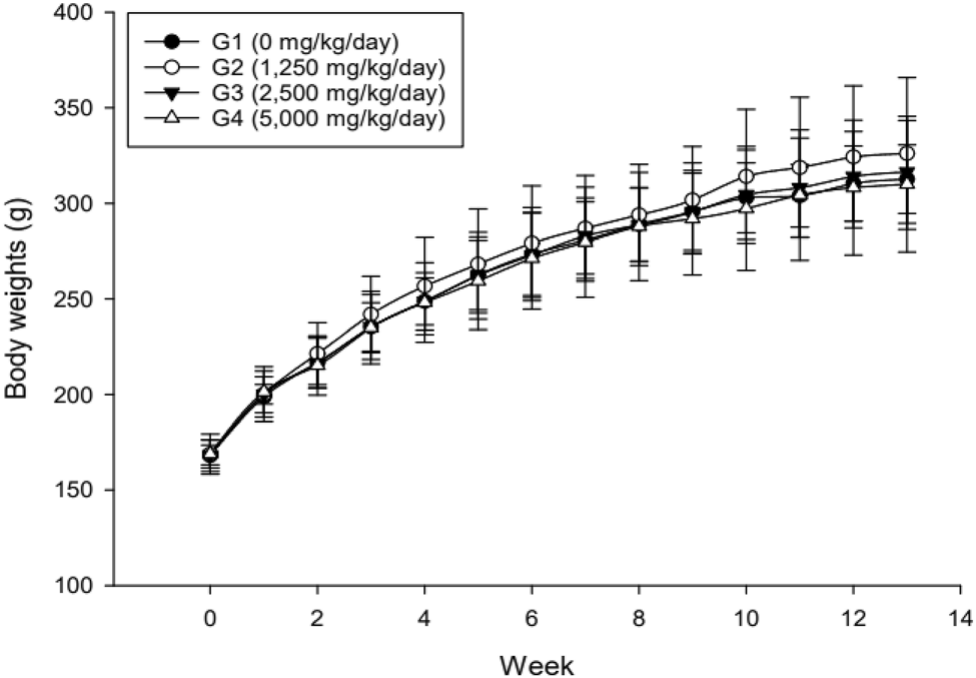
Body weights of female rats in 13-week study of *W. cibaria* CMU. Individual animal weight was recorded at acquisition, grouping, at the before administration, once a week during the study and before necropsy. There was no effect of the test material on female body weight

There were no test substance-related mortalities or abnormal clinical observations. No abnormalities were found in the ophthalmological examination and there were no effects of the test substance on estrus cycle. Body weights of males in the 2500 and 5000 mg/kg bw/day groups were less than controls at week 4, all groups of treated males exhibited lower body weights than controls from weeks 5–8, and males in the 2500 mg/kg bw/day also had lower body weights than controls at week 12 (Fig. 1). Reductions in food consumption in males in the 2500 and 5000 mg/kg bw/day groups were noted compared to controls throughout the study, and a few excursions from control were noted in the 2500 mg/kg bw/day male group (Table 2). These results are considered to be related to administration of the test substance; however, they are not adverse because they are within 10% of the vehicle control group. There was no effect of the test material on female body weight or food consumption (Fig. 2; Table 2).

**Table 2:** Mean food consumption of male and female rats during the 13-week study with *W. cibaria* CMU

Week of Study	Control	1250 mg/kg bw/day	2500 mg/kg bw/day	5000 mg/kg bw/day
Males				
0	21.69 ± 3.30	23.65 ± 1.36	21.31 ± 0.99	23.55 ± 1.22
1	27.79 ± 1.88	26.46 ± 1.76	25.56 ± 1.17	24.84 ± 1.85
2	30.88 ± 1.44	28.38 ± 2.27	28.17 ± 3.00	25.34 ± 2.61^*^
3	30.87 ± 3.53	27.78 ± 3.72	27.13 ± 0.71	26.22 ± 2.39
4	31.17 ± 0.75	27.97 ± 3.48^*^	26.96 ± 1.36^*^	25.80 ± 2.72^*^
5	30.61 ± 1.34	28.02 ± 2.72	27.96 ± 3.08	25.29 ± 2.05^*^
6	31.08 ± 1.72	27.78 ± 4.68	24.95 ± 0.86^**^	24.84 ± 2.20^**^
7	29.76 ± 2.34	27.38 ± 3.30	25.79 ± 1.79^*^	24.73 ± 2.43^*^
8	30.37 ± 1.76	27.35 ± 2.94^**^	26.50 ± 1.64^**^	24.56 ± 1.75^**^
9	28.33 ± 1.83	27.10 ± 2.71	23.36 ± 2.64	27.42 ± 5.07
10	29.81 ± 1.94	28.79 ± 3.71	25.85 ± 2.29	24.55 ± 3.08^*^
11	31.04 ± 1.23	28.26 ± 3.66	25.14 ± 1.21^**^	24.39 ± 2.59^**^
12	30.96 ± 1.88	27.52 ± 3.02^**^	25.64 ± 0.66^**^	23.89 ± 2.03^**^
13	28.73 ± 1.74	25.43 ± 2.04^*^	25.06 ± 1.47^*^	23.60 ± 2.99^*^
Females				
0	14.46 ± 2.14	14.58 ± 2.82	12.83 ± 3.31	13.28 ± 2.37
1	14.50 ± 1.39	15.86 ± 1.16	15.14 ± 3.00	13.87 ± 1.63
2	19.96 ± 2.75	18.51 ± 2.50	18.04 ± 4.27	16.50 ± 3.00
3	20.25 ± 4.36	19.94 ± 2.68	19.42 ± 0.81	20.08 ± 3.84
4	17.99 ± 3.35	20.05 ± 3.63	18.81 ± 1.42	17.93 ± 3.10
5	19.21 ± 2.53	17.90 ± 2.08	19.76 ± 2.40	16.83 ± 2.79
6	20.47 ± 3.67	20.11 ± 4.26	18.67 ± 3.94	17.82 ± 1.68
7	18.16 ± 2.58	20.97 ± 3.21	20.23 ± 2.03	18.31 ± 2.65
8	19.12 ± 3.30	20.31 ± 3.48	18.34 ± 1.96	18.42 ± 3.04
9	18.15 ± 1.10	18.67 ± 3.05	17.96 ± 1.88	15.76 ± 1.89
10	18.13 ± 2.74	19.07 ± 3.10	19.21 ± 2.74	16.32 ± 1.85
11	18.05 ± 1.38	18.65 ± 2.97	16.69 ± 2.40	16.59 ± 1.77
12	17.76 ± 2.42	17.94 ± 2.31	18.87 ± 2.23	15.10 ± 2.98
13	19.41 ± 0.88	18.66 ± 2.36	19.01 ± 3.22	15.48 ± 2.26

N = 5/group. Data are presented as mean ± standard deviation

^*^Significant difference compared with the control group value, *p* < 0.05

^**^Significant difference compared with the control group value, *p* < 0.01

A few changes in hematology, coagulation and clinical chemistry were noted in the study (Table 3, 4). WBC and PT were decreased in males receiving 1250 or 5000 mg/kg bw/day, EOP was decreased in females receiving 1250 or 2500 mg/kg bw/day (however there was no effect on the EOS) and MCHC was decreased in females receiving 2500 mg/kg bw/day; there were no other statistically significant changes in any other hematological parameters (Table 3). BA and BIL of females in the 2500 mg/kg bw/day group were increased compared to the vehicle control group (Table 4). The study investigators did not consider the changes to be toxicologically relevant due to lack of dose response-relationship and biologically insignificant degree of change.

**Table 3:** Hematology and coagulation data for the 13-week study of *W. cibaria* CMU

Parameter	Control	1250 mg/kg bw/day	2500 mg/kg bw/day	5000 mg/kg bw/day
Males				
WBC (10^3^/µL)	7.47 ± 1.00	6.15 ± 1.19^*^	7.30 ± 1.89	6.11 ± 1.03^*^
NEU (10^3^/µL)	1.45 ± 0.43	1.04 ± 0.27	1.41 ± 0.58	1.14 ± 0.36
EOS (10^3^/µL)	0.09 ± 0.03	0.09 ± 0.03	0.09 ± 0.03	0.07 ± 0.01
BAS (10^3^/µL)	0.00 ± 0.00	0.00 ± 0.00	0.00 ± 0.00	0.00 ± 0.00
LYM (10^3^/µL)	5.70 ± 0.71	4.87 ± 1.05	5.60 ± 1.52	4.69 ± 0.91
MON (10^3^/µL)	0.19 ± 0.07	0.12 ± 0.04	0.17 ± 0.05	0.17 ± 0.08
LUC (10^3^/µL)	0.04 ± 0.01	0.02 ± 0.01	0.04 ± 0.02	0.04 ± 0.03
RBC (10^6^/µL)	8.36 ± 0.40	8.57 ± 0.38	8.46 ± 0.23	8.48 ± 0.36
HGB (g/dL)	15.0 ± 0.7	15.1 ± 0.6	15.0 ± 0.3	15.2 ± 0.8
RDW (%)	13.0 ± 0.9	12.5 ± 0.9	12.3 ± 0.4	12.2 ± 0.2
HCT (%)	44.2 ± 1.7	44.5 ± 1.5	44.2 ± 1.0	44.9 ± 2.3
MCV (fL)	53.0 ± 1.2	51.9 ± 1.0	52.3 ± 1.3	53.0 ± 1.0
MCH (pg)	18.0 ± 0.6	17.7 ± 0.4	17.7 ± 0.5	17.9 ± 0.4
MCHC (g/dL)	34.0 ± 0.6	34.0 ± 0.4	34.0 ± 0.3	33.7 ± 0.4
RET (10^3^/µL)	2.27 ± 0.55	1.95 ± 0.40	2.09 ± 0.38	1.85 ± 0.23
MetHGB (%)	1.6 ± 0.8	1.9 ± 0.8	2.1 ± 0.5	1.7 ± 1.0
PLT (10^3^/µL)	1077 ± 86	1012 ± 124	979 ± 146	985 ± 89
MPV (fL)	8.8 ± 0.6	8.9 ± 0.4	8.6 ± 0.3	8.9 ± 0.4
PT (s)	10.91 ± 0.59	9.94 ± 0.46^*^	10.67 ± 1.26	10.04 ± 0.53^*^
APTT (s)	16.6 ± 1.2	16.1 ± 1.1	17.0 ± 1.0	16.8 ± 1.5
Females				
WBC (10^3^/µL)	3.97 ± 1.26	4.88 ± 1.17	4.73 ± 1.65	4.37 ± 1.65
NEU (10^3^/µL)	0.64 ± 0.22	0.66 ± 0.20	0.67 ± 0.25	0.70 ± 0.42
EOS (10^3^/µL)	0.08 ± 0.02	0.06 ± 0.02	0.07 ± 0.02	0.07 ± 0.01
EOP (%)	1.9 ± 0.4	1.2 ± 0.3^**^	1.5 ± 0.5^**^	1.6 ± 0.4
BAS (10^3^/µL)	0.00 ± 0.00	0.00 ± 0.00	0.00 ± 0.00	0.00 ± 0.00
LYM (10^3^/µL)	3.15 ± 1.09	4.02 ± 1.05	3.83 ± 1.49	3.49 ± 1.29
MON (10^3^/µL)	0.09 ± 0.04	0.10 ± 0.05	0.12 ± 0.05	0.10 ± 0.05
LUC (10^3^/µL)	0.02 ± 0.01	0.03 ± 0.02	0.03 ± 0.02	0.02 ± 0.01
RBC (10^6^/µL)	7.73 ± 0.28	7.64 ± 0.37	7.74 ± 0.25	7.61 ± 0.39
HGB (g/dL)	14.6 ± 0.7	14.6 ± 0.6	14.3 ± 0.5	14.2 ± 0.6
RDW (%)	11.1 ± 0.4	11.4 ± 0.4	11.0 ± 0.3	11.4 ± 0.3
HCT (%)	42.5 ± 1.7	42.7 ± 1.1	42.9 ± 1.7	42.6 ± 1.1
MCV (fL)	55.1 ± 1.6	56.0 ± 1.9	55.5 ± 1.4	56.1 ± 3.0
MCH (pg)	18.9 ± 0.5	19.1 ± 0.8	18.5 ± 0.6	18.7 ± 1.1
MCHC (g/dL)	34.4 ± 0.6	34.2 ± 0.8	33.4 ± 0.6^*^	33.4 ± 1.2
RET (10^3^/µL)	2.1 ± 0.3	2.2 ± 0.4	2.0 ± 0.5	2.1 ± 0.2
MetHGB (%)	1.4 ± 0.9	1.4 ± 0.9	1.5 ± 0.7	1.7 ± 0.9
PLT (10^3^/µL)	1019 ± 90	972 ± 88	1017 ± 78	971 ± 98
MPV (fL)	8.7 ± 0.6	8.9 ± 0.3	8.4 ± 0.5	8.8 ± 0.4
PT (s)	9.32 ± 0.56	9.15 ± 0.68	9.25 ± 0.51	9.31 ± 0.59
APTT (s)	17.3 ± 0.6	17.3 ± 1.2	17.7 ± 1.6	17.1 ± 1.1

*APTT* activated partial thromboplastin time, *BAS* basophils, *dL* deciliter, *EOP* percentage of eosinophils, *EOS* eosinophils, *fL* femtoliter, *g* gram, *HCT* hematocrit, *HGB* hemoglobin concentration, *kg* kilogram, *LUC* large unstained cells, *LYM* lymphocytes, *metHGB* methemoglobin, *MCH* mean corpuscular hemoglobin, *MCHC* mean corpuscular hemoglobin concentration, *MCV* mean corpuscular volume, *mg* milligrams, *MON* monocytes, *MPV* mean platelet volume, *NEU* neutrophils, *pg *picograms, *PLT* platelets, *PT* prothrombin time, *RBC* erythrocytes, *RDW* red blood cell distribution width, *RET* reticulocytes, *s* seconds, *WBC* white blood cells (leukocytes), *µL* microliter

N = 10/group. Data are presented as mean ± standard deviation. Differential counts for WBC are also reported as percentages if any of the values are significantly different from the control group

^*^Significant difference compared with the control group value, *p* < 0.05

^**^Significant difference compared with the control group value, *p* < 0.01

**Table 4:** Clinical chemistry data for the 13-week study of *W. cibaria* CMU

Parameter	Control	1250 mg/kg bw/day	2500 mg/kg bw/day	5000 mg/kg bw/day
Males				
AST (IU/L)	128 ± 16	126 ± 17	129 ± 25	122 ± 29
ALT (IU/L)	34 ± 5	32 ± 7	32 ± 5	30 ± 5
ALP (IU/L)	249 ± 43	261 ± 54	257 ± 55	262 ± 54
BA (µmol/L)	12.3 ± 6.8	11.7 ± 4.6	28.6 ± 53.1	15.1 ± 9.5
BIL (mg/dL)	0.05 ± 0.02	0.05 ± 0.01	0.05 ± 0.05	0.04 ± 0.02
BUN (mg/dL)	13.7 ± 1.2	14.5 ± 1.9	13.6 ± 2.7	14.8 ± 2.7
CRE (mg/dL)	0.43 ± 0.05	0.44 ± 0.04	0.44 ± 0.03	0.46 ± 0.05
UA (mg/dl)	1.0 ± 0.2	1.0 ± 0.1	0.9 ± 0.1	0.9 ± 0.1
GLU (mg/dL)	151 ± 22	152 ± 21	149 ± 19	155 ± 32
CHO (mg/dL)	72 ± 17	78 ± 16	74 ± 21	68 ± 9
HDL (mg/dL)	20 ± 4	22 ± 3	22 ± 4	21 ± 2
LDL (mg/dL)	7 ± 2	8 ± 2	8 ± 2	7 ± 1
TG (mg/dL)	52 ± 24	54 ± 21	51 ± 28	80 ± 38
PRO (g/dL)	6.1 ± 0.3	6.2 ± 0.3	6.1 ± 0.4	6.2 ± 0.3
ALB (g/dL)	2.3 ± 0.1	2.3 ± 0.1	2.3 ± 0.1	2.3 ± 0.1
ALB/GLOB	0.60 ± 0.03	0.59 ± 0.03	0.62 ± 0.05	0.60 ± 0.04
LDH (IU/L)	1139 ± 364	1260 ± 394	1132 ± 366	1169 ± 727
CPK (U/L)	527 ± 157	563 ± 185	542 ± 189	491 ± 278
ChE (U/L)	180 ± 67	161 ± 49	149 ± 42	180 ± 45
Ca (mg/dL)	9.8 ± 0.4	9.8 ± 0.5	9.8 ± 0.7	10.1 ± 0.6
IP (mg/dL)	6.6 ± 0.6	6.5 ± 0.6	6.8 ± 0.4	6.8 ± 0.9
Mg (mg/dL)	2.6 ± 0.3	2.6 ± 0.4	2.6 ± 0.3	2.7 ± 0.3
Na (mmol/L)	144 ± 1	144 ± 1	144 ± 1	144 ± 1
K (mmol/L)	4.9 ± 0.1	4.9 ± 0.1	4.8 ± 0.2	4.8 ± 0.2
Cl (mmol/L)	102 ± 2	103 ± 1	104 ± 2	102 ± 1
TSH (ng/mL)	1.944 ± 0.855	1.586 ± 0.377	1.396 ± 0.526	1.449 ± 0.455
T3 (ng/mL)	0.466 ± 0.090	0.448 ± 0.129	0.426 ± 0.071	0.494 ± 0.075
T4 (ng/mL)	45.686 ± 3.819	45.856 ± 4.253	44.546 ± 4.647	45.537 ± 2.518
Females				
AST (IU/L)	119 ± 16	112 ± 27	115 ± 16	110 ± 22
ALT (IU/L)	30 ± 5	31 ± 17	29 ± 5	27 ± 6
ALP (IU/L)	141 ± 44	131 ± 24	130 ± 47	134 ± 33
BA (µmol/L)	13.5 ± 5.1	12.9 ± 4.2	19.2 ± 6.8^*^	13.9 ± 4.6
BIL (mg/dL)	0.08 ± 0.02	0.09 ± 0.02	0.11 ± 0.03^*^	0.08 ± 0.02
BUN (mg/dL)	16.5 ± 3.9	16.6 ± 1.8	15.1 ± 1.4	16.5 ± 2.3
CRE (mg/dL)	0.53 ± 0.06	0.52 ± 0.05	0.50 ± 0.03	0.52 ± 0.05
UA (mg/dl)	0.7 ± 0.1	0.8 ± 0.1	0.8 ± 0.2	0.8 ± 0.2
GLU (mg/dL)	147 ± 26	151 ± 22	151 ± 25	139 ± 15
CHO (mg/dL)	87 ± 12	88 ± 19	86 ± 15	83 ± 17
HDL (mg/dL)	28 ± 2	27 ± 4	27 ± 3	27 ± 4
LDL (mg/dL)	5 ± 1	6 ± 1	5 ± 1	5 ± 1
TG (mg/dL)	38 ± 13	45 ± 36	41 ± 21	40 ± 25
PRO (g/dL)	6.8 ± 0.5	7.0 ± 0.5	6.9 ± 0.5	6.8 ± 0.3
ALB (g/dL)	2.9 ± 0.3	3.1 ± 0.3	3.0 ± 0.3	2.9 ± 0.2
ALB/GLOB	0.76 ± 0.06	0.78 ± 0.09	0.78 ± 0.03	0.75 ± 0.06
LDH (IU/L)	712 ± 284	734 ± 282	762 ± 336	751 ± 382
CPK (U/L)	379 ± 154	338 ± 124	378 ± 179	392 ± 196
ChE (U/L)	1729 ± 814	1449 ± 278	1558 ± 367	1602 ± 306
Ca (mg/dL)	9.9 ± 0.5	10.0 ± 0.5	10.0 ± 0.4	10.0 ± 0.4
IP (mg/dL)	5.7 ± 0.7	5.5 ± 0.7	5.4 ± 0.6	5.9 ± 0.7
Mg (mg/dL)	2.7 ± 0.3	2.6 ± 0.3	2.8 ± 0.2	2.7 ± 0.3
Na (mmol/L)	143 ± 2	144 ± 2	144 ± 2	144 ± 2
K (mmol/L)	4.4 ± 0.2	4.2 ± 0.2	4.2 ± 0.2	4.3 ± 0.2
Cl (mmol/L)	103 ± 1	102 ± 3	101 ± 2	102 ± 2
TSH (ng/mL)	1.925 ± 1.227	1.667 ± 0.560	1.614 ± 0.757	1.485 ± 0.743
T3 (ng/mL)	0.642 ± 0.119	0.585 ± 0.132	0.598 ± 0.189	0.552 ± 0.215
T4 (ng/mL)	42.115 ± 4.110	41.548 ± 4.580	41.603 ± 4.754	42.452 ± 4.595

*ALB* albumin, *ALP* alkaline phosphatase, *ALT* alanine aminotransferase, *AST* aspartate aminotransferase, *BA* bile acid, *BIL* total bilirubin, *BUN* blood urea nitrogen, *Ca* calcium, *ChE* cholinesterase, *CHO* cholesterol, *Cl* chloride, *CPK* creatine phosphokinase, *CRE* creatinine, *dL* deciliter, *g* grams, *ALB/GLOB* albumin/globulin ratio, *GLU* glucose, *HDL* high density lipoprotein cholesterol, *IP* inorganic phosphorus,* IU* international units, *K* potassium, *L* liter, *LDH* lactate dehydrogenase, *LDL* low density lipoprotein cholesterol, *Mg* magnesium, *mg* milligrams, *mL* milliliters, *mmol* millimoles, *Na* sodium, *ng* nanograms, *PRO* total protein, *TG* triglycerides, *TSH* thyroid stimulating hormone, *T4* thyroxine, *T3* tri-iodothyronine, *U* units, *UA* uric acid, *µmol* micromole

N = 10/group. Data are presented as mean ± standard deviation

^*^Significant difference compared with the control group value, *p* < 0.05

Administration of the test material was associated with some changes in urinalysis (Table 5). The specific gravity of the male test groups and pH levels of the male and female test groups showed dose-dependent increases. The urine volume of the male 5000 mg/kg/day test group also was lower than the vehicle control group. The effects on urinalysis were not considered by the study investigators to be toxicological effects because no abnormal changes in clinical pathology and histopathology related to the kidney were observed.

**Table 5:** Urinalysis of male and female rats given *W. cibaria* CMU for 13-weeks

Parameter	Result	Control	1250 mg/kg bw/day	2500 mg/kg bw/day	5000 mg/kg bw/day
Males					
Urine volume (mL)		24 ± 9	24 ± 5	18 ± 2	12 ± 3^*^
Glucose	Negative	5/5	5/5	5/5	5/5
Bilirubin	Negative	5/5	5/5	5/5	5/5
Ketone bodies	Negative	5/5	5/5	5/5	3/5
	Trace	0/5	0/5	0/5	2/5
Specific gravity^*^	≤ 1.005	3/5	5/5	3/5	0/5
	1.010	2/5	0/5	2/5	5/5
Occult blood	Negative	3/5	3/5	5/5	3/5
	Trace	2/5	2/5	0/5	2/5
pH^*^	7.5	1/5	3/5	0/5	1/5
	8.0	3/5	2/5	5/5	0/5
	8.5	1/5	0/5	0/5	3/5
	9.0	0/5	0/5	0/5	1/5
Protein	Negative	5/5	5/5	5/5	3/5
	Trace	0/5	0/5	0/5	2/5
Urobilinogen	0.2	5/5	5/5	5/5	5/5
Nitrite	Negative	5/5	5/5	5/5	5/5
Leukocytes	Negative	4/5	5/5	5/5	4/5
	Trace	0/5	0/5	0/5	1/5
	1 +	1/5	0/5	0/5	0/5
Color	Straw	5/5	5/5	5/5	5/5
Females					
Urine volume (mL)		11 ± 4	12 ± 4	11 ± 2	11 ± 3
Glucose	Negative	5/5	5/5	5/5	5/5
Bilirubin	Negative	5/5	5/5	5/5	5/5
Ketone bodies	Negative	5/5	5/5	5/5	5/5
Specific gravity	≤ 1.005	5/5	5/5	3/5	3/5
	1.010	0/5	0/5	2/5	2/5
Occult blood	Negative	4/5	5/5	4/5	4/5
	Trace	1/5	0/5	1/5	0/5
	1 +	0/5	0/5	0/5	1/5
pH^**^	7.0	2/5	0/5	0/5	1/5
	7.5	2/5	3/5	0/5	1/5
	8.0	1/5	2/5	5/5	0/5
	8.5	0/5	0/5	0/5	3/5
Protein	Negative	5/5	5/5	5/5	5/5
Urobilinogen	0.2	5/5	5/5	5/5	5/5
Nitrite	Negative	5/5	5/5	5/5	5/5
Leukocytes	Negative	5/5	5/5	5/5	5/5
Color	Straw	5/5	5/5	5/5	5/5

Results are presented as number of animals with the sign/number of animals examined for all variables except urine volume, which is presented as mean ± standard deviation

^*^Significant difference among groups, *p* < 0.05

^**^Significant difference among groups, *p* < 0.01. Individual groups were not compared to controls

No abnormal gross findings were noted at necropsy (Table 6). A few changes in organ weights were observed: the left side adrenal gland weight (absolute) of males receiving 1250 mg/kg/day was decreased compared to control, as well the right-side thyroid weight (absolute) of males receiving 2500 mg/kg bw/day. Paradoxically, the right-side thyroid weight (absolute and relative to body weight) of males receiving 5000 mg/kg bw/day was increased with respect to control. The study investigators did not consider the findings to be toxicologically relevant based on lack of dose–response relationship or corresponding histopathological findings. As shown in Table 4, there was no effect of the test substance on TSH, T3 or T4 in males or females.

**Table 6:** Absolute organ weights (g) and relative organ to body weights (%) in 13-week study of *W. cibaria* CMU

Parameter	Control	1250 mg/kg bw/day	2500 mg/kg bw/day	5000 mg/kg bw/day
Males				
Terminal Body weight (g)	596.32 ± 33.53	569.19 ± 58.52	548.93 ± 27.18	563.02 ± 35.76
Testis L (g)	1.8903 ± 0.1517	1.9193 ± 0.2019	1.8525 ± 0.1933	1.8433 ± 0.1221
Testis R (g)	1.8808 ± 0.1390	1.9307 ± 0.1780	1.8567 ± 0.1731	1.8337 ± 0.1196
Testis L/TBW (%)	0.3179 ± 0.0316	0.3410 ± 0.0533	0.3372 ± 0.0274	0.3291 ± 0.0348
Testis R/TBW (%)	0.3162 ± 0.0286	0.3432 ± 0.0525	0.3383 ± 0.0278	0.3272 ± 0.0332
Epididymis L (g)	0.8297 ± 0.1189	0.7730 ± 0.0879	0.7446 ± 0.1336	0.7742 ± 0.0705
Epididymis R (g)	0.8007 ± 0.0999	0.7802 ± 0.0964	0.7430 ± 0.1495	0.7781 ± 0.0589
Epididymis L/TBW (%)	0.1396 ± 0.0222	0.1371 ± 0.0211	0.1354 ± 0.0218	0.1384 ± 0.0181
Epididymis R/TBW (%)	0.1347 ± 0.0183	0.1385 ± 0.0240	0.1350 ± 0.0238	0.1389 ± 0.0159
Prostate (g)	3.7147 ± 0.4305	3.8087 ± 0.6243	3.5114 ± 0.5642	3.7407 ± 0.3639
Prostate/TBW (%)	0.6250 ± 0.0821	0.6694 ± 0.0920	0.6398 ± 0.1025	0.6672 ± 0.0826
Spleen (g)	0.9774 ± 0.1611	0.8671 ± 0.0846	0.9365 ± 0.1192	0.8886 ± 0.1070
Spleen/TBW (%)	0.1637 ± 0.0246	0.1527 ± 0.0090	0.1710 ± 0.0250	0.1577 ± 0.0142
Liver (g)	14.8266 ± 1.3481	14.4260 ± 1.9448	13.4434 ± 2.0199	14.3966 ± 1.8601
Liver/TBW (%)	2.4860 ± 0.1660	2.5296 ± 0.1750	2.4427 ± 0.3192	2.5510 ± 0.2092
Adrenal gland L (g)	0.0355 ± 0.0044	0.0305 ± 0.0048^*^	0.0321 ± 0.0043	0.0350 ± 0.0042
Adrenal gland R (g)	0.0335 ± 0.0036	0.0289 ± 0.0037	0.0312 ± 0.0051	0.0322 ± 0.0045
Adrenal gland L/TBW (%)	0.0060 ± 0.0009	0.0054 ± 0.0009	0.0059 ± 0.0009	0.0062 ± 0.0009
Adrenal gland R/TBW (%)	0.0056 ± 0.0008	0.0051 ± 0.0008	0.0057 ± 0.0010	0.0057 ± 0.0009
Kidney L (g)	1.8931 ± 0.2003	1.7530 ± 0.2371	1.7611 ± 0.0897	1.7417 ± 0.1067
Kidney R (g)	1.8950 ± 0.1663	1.7790 ± 0.2270	1.7737 ± 0.0814	1.7014 ± 0.0999
Kidney L/TBW (%)	0.3180 ± 0.0343	0.3089 ± 0.0360	0.3216 ± 0.0238	0.3100 ± 0.0199
Kidney R/TBW (%)	0.3183 ± 0.0294	0.3130 ± 0.0317	0.3239 ± 0.0239	0.3029 ± 0.0208
Heart (g)	1.7376 ± 0.1153	1.6805 ± 0.1075	1.6639 ± 0.1789	1.7088 ± 0.1927
Heart/TBW	0.2917 ± 0.0178	0.2967 ± 0.0196	0.3044 ± 0.0433	0.3031 ± 0.0217
Lung (g)	1.9597 ± 0.0784	1.8758 ± 0.2577	1.8585 ± 0.1358	1.8537 ± 0.1548
Lung/TBW (%)	0.3290 ± 0.0115	0.3308 ± 0.0426	0.3392 ± 0.0294	0.3298 ± 0.0265
Brain (g)	2.1955 ± 0.1095	2.2147 ± 0.0631	2.2163 ± 0.0797	2.2033 ± 0.0908
Brain/TBW (%)	0.3693 ± 0.0285	0.3929 ± 0.0428	0.4047 ± 0.0267	0.3922 ± 0.0205
Pituitary (g)	0.0141 ± 0.0019	0.0142 ± 0.0029	0.0144 ± 0.0016	0.0143 ± 0.0015
Pituitary/TBW (%)	0.0024 ± 0.0003	0.0025 ± 0.0005	0.0026 ± 0.0002	0.0025 ± 0.0002
Thymus (g)	0.3693 ± 0.0928	0.3139 ± 0.0698	0.3036 ± 0.0948	0.3138 ± 0.0692
Thymus/TBW (%)	0.0620 ± 0.0150	0.0551 ± 0.0110	0.0550 ± 0.0153	0.0555 ± 0.0100
Thyroid gland L (g)	0.0107 ± 0.0054	0.0091 ± 0.0018	0.0079 ± 0.0019	0.0108 ± 0.0018
Thyroid gland R (g)	0.0103 ± 0.0035	0.0087 ± 0.0023	0.0073 ± 0.0023^**^	0.0130 ± 0.0027^**^
Thyroid gland L/TBW (%)	0.0018 ± 0.0009	0.0016 ± 0.0003	0.0014 ± 0.0004	0.0019 ± 0.0004
Thyroid gland R/TBW (%)	0.0017 ± 0.0006	0.0015 ± 0.0004	0.0013 ± 0.0004	0.0023 ± 0.0005^**^
Females				
Terminal Body weight (g)	296.99 ± 17.51	308.85 ± 35.51	300.47 ± 25.96	295.11 ± 34.27
Ovary L (g)	0.0480 ± 0.0105	0.0510 ± 0.0110	0.0531 ± 0.0103	0.0467 ± 0.0113
Ovary R (g)	0.0524 ± 0.0145	0.0431 ± 0.0127	0.0541 ± 0.0121	0.0485 ± 0.0100
Ovary L/TBW (%)	0.0161 ± 0.0032	0.0165 ± 0.0026	0.0177 ± 0.0030	0.0161 ± 0.0047
Ovary R/TBW (%)	0.0176 ± 0.0047	0.0138 ± 0.0030	0.0180 ± 0.0037	0.0165 ± 0.0036
Uterus (g)	0.6149 ± 0.1251	0.6894 ± 0.1723	0.7100 ± 0.2488	0.6639 ± 0.2877
Uterus/TBW (%)	0.2085 ± 0.0487	0.2272 ± 0.0685	0.2340 ± 0.0667	0.2275 ± 0.1007
Spleen (g)	0.5402 ± 0.0833	0.5896 ± 0.1332	0.5531 ± 0.0614	0.5208 ± 0.0367
Spleen/TBW (%)	0.1821 ± 0.0279	0.1908 ± 0.0377	0.1842 ± 0.0141	0.1779 ± 0.0177
Liver (g)	7.5055 ± 0.7752	8.0649 ± 1.1943	7.7133 ± 0.6764	7.6137 ± 1.0426
Liver/TBW (%)	2.5254 ± 0.1907	2.6068 ± 0.1954	2.5721 ± 0.1702	2.5768 ± 0.1584
Adrenal gland L (g)	0.0361 ± 0.0079	0.0399 ± 0.0065	0.0399 ± 0.0065	0.0376 ± 0.0049
Adrenal gland R (g)	0.0358 ± 0.0068	0.0377 ± 0.0093	0.0403 ± 0.0089	0.0355 ± 0.0082
Adrenal gland L/TBW (%)	0.0121 ± 0.0025	0.0129 ± 0.0016	0.0133 ± 0.0018	0.0128 ± 0.0013
Adrenal gland R/TBW (%)	0.0120 ± 0.0022	0.0121 ± 0.0021	0.0134 ± 0.0025	0.0120 ± 0.0024
Kidney L (g)	0.9466 ± 0.1035	1.0345 ± 0.1077	1.0091 ± 0.1281	0.9419 ± 0.0857
Kidney R (g)	0.9798 ± 0.1142	1.0475 ± 0.1179	1.0429 ± 0.1414	0.9811 ± 0.0853
Kidney L/TBW (%)	0.3185 ± 0.0259	0.3357 ± 0.0168	0.3354 ± 0.0267	0.3209 ± 0.0271
Kidney R/TBW (%)	0.3293 ± 0.0246	0.3396 ± 0.0171	0.3464 ± 0.0291	0.3340 ± 0.0244
Heart (g)	1.0590 ± 0.1552	1.1169 ± 0.1397	1.1229 ± 0.1202	0.9995 ± 0.0819
Heart/TBW	0.3573 ± 0.0536	0.3622 ± 0.0260	0.3746 ± 0.0375	0.3404 ± 0.0242
Lung (g)	1.2936 ± 0.0883	1.3849 ± 0.1414	1.3990 ± 0.1218	1.3013 ± 0.1062
Lung/TBW (%)	0.4360 ± 0.0255	0.4498 ± 0.0312	0.4666 ± 0.0320	0.4439 ± 0.0422
Brain (g)	1.9838 ± 0.1113	1.9994 ± 0.0955	1.9947 ± 0.1002	2.0042 ± 0.0886
Brain/TBW (%)	0.6693 ± 0.0446	0.6529 ± 0.0595	0.6665 ± 0.0414	0.6857 ± 0.0699
Pituitary (g)	0.0185 ± 0.0017	0.0197 ± 0.0057	0.0229 ± 0.0058	0.0185 ± 0.0022
Pituitary/TBW (%)	0.0062 ± 0.0005	0.0064 ± 0.0017	0.0077 ± 0.0022	0.0064 ± 0.0010
Thymus (g)	0.2755 ± 0.0539	0.3151 ± 0.0732	0.3148 ± 0.0734	0.2617 ± 0.0435
Thymus/TBW (%)	0.0924 ± 0.0145	0.1017 ± 0.0197	0.1041 ± 0.0184	0.0896 ± 0.0172
Thyroid gland L (g)	0.0061 ± 0.0031	0.0064 ± 0.0026	0.0073 ± 0.0027	0.0078 ± 0.0035
Thyroid gland R (g)	0.0058 ± 0.0031	0.0068 ± 0.0030	0.0080 ± 0.0022	0.0077 ± 0.0042
Thyroid gland L/TBW (%)	0.0021 ± 0.0011	0.0021 ± 0.0008	0.0024 ± 0.0007	0.0026 ± 0.0009
Thyroid gland R/TBW (%)	0.0020 ± 0.0011	0.0021 ± 0.0008	0.0026 ± 0.0006	0.0025 ± 0.0011

*g* grams, *L* left, *R* right, *TBW* terminal body weight

N = 10/group. Data are presented as mean ± standard deviation

^*^Significant difference compared with the control group value, *p* < 0.05

^**^Significant difference compared with the control group value, *p* < 0.01

Histopathological lesions such as necrosis of the liver, cell infiltration, mononuclear in lung, kidney and harderian gland, cortical scar in kidney, cystic degeneration in adrenal gland, atrophy, acinar cell in pancreas, ectopic thymus and ultimobranchial cyst in thyroid, and cardiomyopathy in heart were observed in control or high dose animals (Table 7). The lesions were considered to be incidental or spontaneous due to similar incidences in the two groups.

**Table 7:** Histopathological lesions noted in the 13-week study with *W. cibaria* CMU

Parameter	Control	5000 mg/kg bw/day
Males		
Thyroid (Ultimobranchial cyst)	3/10	5/10
Lung (Cell infiltration, inflammatory, focal, bronchiolar/alveolar, minimal)	1/10	0/10
Heart (Cardiomyopathy, minimal)	0/10	1/10
Females		
Liver (Necrosis, focal, mild)	1/10	1/10
Kidney (Cell infiltration, mononuclear, focal, cortex, minimal)	1/10	0/10
Kidney (Cortical scar, minimal)	1/10	0/10
Adrenal gland (Cystic degeneration, focal, zona fasciculata, minimal)	1/10	0/10
Pancreas (Atrophy, acinar, focal, minimal)	1/10	0/10
Thyroid (Ultimobranchial cyst)	3/10	2/10
Thyroid (Ectopic thymus)	1/10	0/10
Harderian gland (Cell infiltration, mononuclear, focal, minimal)	1/10	0/10

Results are depicted as incidences. No remarkable lesions were reported in other organs examined

### Bacterial reverse mutagenic assay with *W. cibaria* CMU

As shown in Table 8, *W. cibaria* CMU was nonmutagenic in the bacterial reverse mutation assay. It did not induce any biologically significant or dose-dependent increases in the number of revertant colonies in any strain tested in the absence or presence of metabolic activation (i.e., S9 mix). Cytotoxicity was not observed at any concentration and there was no mention of test material precipitation. The assay was valid, as the positive controls showed a distinct increase in revertants meeting the criteria for a positive response.

**Table 8:** Reverse mutation assay of *W. cibaria* CMU—mean number of revertants (colony/plate)

Conc. (µg/plate)	TA100		TA1535		WP2*uvrA*		TA98		TA1537	
	− S9	+ S9	− S9	+ S9	− S9	+ S9	− S9	+ S9	−S9	+ S9
0^a^	65 ± 1.5	68 ± 1.2	9 ± 1.7	10 ± 2.0	28 ± 2.1	34 ± 1.7	16 ± 0.6	22 ± 1.0	5 ± 1.0	5 ± 1.5
62	71 ± 2.1	62 ± 1.0	12 ± 0.6	10 ± 1.0	31 ± 1.0	33 ± 2.0	15 ± 1.5	18 ± 1.5	5 ± 1.2	7 ± 2.3
185	70 ± 1.5	66 ± 1.0	7 ± 0.6	12 ± 2.3	34 ± 1.2	36 ± 1.0	12 ± 1.0	17 ± 1.0	5 ± 0.6	8 ± 1.2
556	70 ± 1.5	64 ± 0.6	11 ± 0.6	9 ± 1.2	30 ± 1.5	35 ± 1.0	12 ± 1.2	20 ± 0.0	5 ± 1.0	8 ± 0.6
1667	70 ± 1.5	65 ± 0.6	9 ± 1.5	9 ± 1.0	29 ± 0.6	37 ± 1.5	15 ± 0.6	20 ± 0.6	5 ± 1.2	5 ± 1.0
5000	68 ± 1.5	64 ± 0.6	10 ± 1.5	10 ± 0.6	35 ± 1.5	35 ± 0.0	18 ± 1.2	18 ± 1.5	7 ± 0.6	6 ± 0.6
PC	431 ± 94.9^b^	439 ± 85.9^c^	344 ± 46.1^d^	261 ± 28.4^c^	269 ± 45.6^e^	244 ± 34.6^c^	403 ± 44.1^b^	416 ± 36.7^f^	2025 ± 33.1^ g^	392 ± 24.0^c^

Substance was tested using the pre-incubation method with and without metabolic activation system (S9). Data are presented as mean ± standard deviation of three replicates per test condition

No significant increases in the number of revertant colonies were observed compared to the negative control

Negative control: ^a^distilled water. Positive controls (PC): ^b^2-(2-furyl)-3–5-nitro-2-furyl) (AF-2); ^c^2-aminoanthracene (2-AA); ^d^sodium azide (NaN3); ^e^4-nitroquinoline 1-oxide (4-NQO); ^f^benzo(a)pyrene (BP); ^g^9-aminoacridine (9-AA). Conc. = concentration

### In vitro chromosome aberration assay with *W. cibaria* CMU

Test concentrations were limited by cytotoxicity to 20.58, 61.73 and 185.19 µg/mL in the 24 h study, 61.73, 185.19 and 555.56 µg/mL in 6 h study, and 185.19, 555.56, 1666.67 µg/mL in the 6 h study + S9 (Table 9). There was no effect of the test substance on the mean number of chromosome aberrations observed of the number of cells with chromosome aberrations under any condition. The test substance also did not induce a statistically significant increase in the number of cells with polyploidy or endoreduplication compared to the negative control under any condition. The positive control induced a statistically significant increase in the number of cells with chromosome aberrations, confirming that the assay had adequate sensitivity. Under the conditions of the study, the test substance did not induce chromosome aberrations in cultured CHO-k1 cells.

**Table 9:** Effect of *W. cibaria* CMU on chromosome aberrations in Chinese Hamster Ovary cells

Conc. (µg/mL)	RICC	Mean number of total CA		Mean number of cells with CA		PP + ER
		(−) Gap	(+) Gap	(−) Gap	(+) Gap	
24 h study − S9						
NC^a^	100	0.33	0.33	0.33	0.33	0
20.58	68.01	1.00	1.00	1.00	1.00	0
61.73	60.39	0.67	0.67	0.67	0.67	0
185.19	46.40	0.33	0.33	0.33	0.33	0
PC^b^	N	24.00	24.00	24.00^*^	24.00	0
6 h study − S9						
NC^a^	100	0.67	0.67	0.67	0.67	0
61.73	71.45	0.67	0.67	0.67	0.67	0
185.19	61.55	0.67	0.67	0.67	0.67	0
555.56	46.77	0.67	0.67	0.67	0.67	0
PC^b^	N	21.67	21.67	21.67^*^	21.67	0
6 h study + S9						
NC^a^	100	0.67	0.67	0.67	0.67	0
185.19	80.69	1.00	1.00	1.00	1.00	0
555.56	61.37	0.67	0.67	0.67	0.67	0
1666.67	49.64	0.67	0.67	0.67	0.67	0
PC^c^	N	25.00	25.00	25.00^*^	25.00	0

*NC* negative control, *PC* positive control, *CA* chromosome aberrations, *ER* endoreduplication, *N* not tested, *PP* polyploidy, *RICC* relative increase in cell counts compared to negative control

^*^Significantly different from control at *p* < 0.05

^a^Distilled water

^b^Mitomycin C (0.04 µg/mL)

^c^Cyclophosphamide (10 µg/mL)

### Mammalian micronucleus assay with *W. cibaria* CMU

Result of the micronucleus study are shown in Table 10. No animal deaths occurred in any of the dose groups in the micronucleus test. There was no effect of the test substance on body weight or PCE/PCE + NCE ratio and no abnormal clinical signs were noted. There also was no effect of any dose of the test material on the incidence of MNPCE/4000 PCE compared to the negative control. Under the conditions of the study, the test substance did not induce the formation of micronucleated PCE in the bone marrow of mice at up to 5000 mg/kg bw/day. The assay was valid as the MN-PCE frequencies for the negative control mice were within the historical range (0.08–0.32%) and an adequate positive control response was obtained (9.52 ± 1.08% MN-PCE).

**Table 10:** In vivo micronucleus test of *W. cibaria* CMU—the frequency of MNPCE in 4000 PCEs and the ratio of PCE

Groups	Dose (mg/kg bw/day)	Animal No.	MNPCE/4,000 PCEs (%)	PCE/(PCE/NCE)
Negative control Distilled water	0	1	0.15	0.58
		2	0.08	0.58
		3	0.10	0.58
		4	0.13	0.59
		5	0.13	0.57
		Mean ± SD	0.12 ± 0.03	0.58 ± 0.01
Test substance *W. cibaria* CMU	1250	6	0.13	0.53
		7	0.08	0.58
		8	0.23	0.55
		9	0.13	0.50
		10	0.13	0.56
		Mean ± SD	0.14 ± 0.05	0.54 ± 0.03
	2500	11	0.15	0.57
		12	0.15	0.59
		13	0.10	0.49
		14	0.08	0.56
		15	0.18	0.54
		Mean ± SD	0.13 ± 0.04	0.55 ± 0.04
	5000	16	0.08	0.56
		17	0.10	0.57
		18	0.08	0.57
		19	0.08	0.48
		20	0.10	0.58
		Mean ± SD	0.09 ± 0.01	0.55 ± 0.04
Positive control Mitomycin C	2	21	9.00	0.47
		22	8.65	0.44
		23	10.23	0.51
		23	10.23	0.51
		24	11.05	0.45
		25	8.65	0.44
		Mean ± SD	9.52 ± 1.08^**^	0.46 ± 0.03^*^

*MNPCE* micronucleated polychromatic erythrocytes, *NCE* normochromatic erythrocytes, *PCE* polychromatic erythrocytes

^*^Significantly different from the control, *p* < 0.05

^**^Significantly different from the control, *p* < 0.01

## Discussion

*W. cibaria* CMU (oraCMU®) is being developed for use in human food and dietary supplements. Results of previous studies indicate that the strain is susceptible to common antibiotics, with the possible exception of kanamycin and vancomycin, but does not transfer resistance of these antibiotics to other bacteria [8]. Genetic analysis confirmed that antibiotic resistance to kanamycin is an intrinsic characteristic of *W. cibaria* and is not unique to *W. cibaria* CMU*.* Additional studies performed by Kang et al. [8] showed that *W. cibaria* CMU does not harbor virulence genes associated with pathogenic bacteria, does not cause hemolysis, mucin or protein degradation, or platelet aggregation, and does not produce d-lactate or urease (which catalyzes the hydrolysis of urea to ammonia). However, animal toxicity or genotoxicity studies on *W. cibaria* strains have not been conducted previously. Results of the studies described herein add to the database of information that *W. cibaria* CMU will be safe for human consumption.

When administered acutely to rats by gavage, up to 5000 mg/kg bw (1.8 × 10^9^ CFU/kg bw), the highest dose administered was well tolerated. This dose was chosen as the highest dose administered in a 14-day range finding and 13-week study in rats. In the 14-day study, changes in MPV of males receiving 2500 and 5000 mg/kg bw/day, NEP in females receiving 1250 and 2500 mg/kg bw/day, and BUN and TG in females receiving 1250 mg/kg bw/day were not considered to be toxicologically relevant due to lack of dose dependency and/or minor nature of the changes. Changes in these variables were not found in rats receiving up to 5000 mg/kg bw (1.8 × 10^9^ CFU/kg bw) *W. cibaria* CMU for ninety days, supporting the conclusion that the findings in the 14-day study were not toxicologically relevant.

The no observed adverse effect level (NOAEL) of the 13-week study is 5000 mg/kg bw/day (1.8 × 10^9^ CFU/kg bw/day), the highest dose administered. In the 13-week study, the most notable effect of administration of the test material was reduced body weight and food consumption of males in the 2500 and 5000 mg/kg bw/day groups. The study investigators did not consider the findings to be adverse because the body weights of the animals were within 10% of the vehicle control group. The 10% criterion is commonly used to determine whether an effect of a test material on body weight is adverse [17]. Changes in a few hematological, coagulation, and clinical chemistry parameters were observed in treated animals; however, the study investigators considered them not to be toxicologically relevant due to lack of dose response-relationship and biologically insignificant degree of change. Regarding the urinalysis, the specific gravity of the male test groups and pH levels of the male and female test groups showed dose-dependent increases. At the 5000 mg/kg bw/dose, the urine volume of males was less than controls. The reason for this is unclear but could be possibly due to decreased water consumption by this group. Because water consumption was not measured, this cannot be confirmed. As noted by the study investigators, no abnormal changes in clinical pathology and histopathology related to the kidney were observed. Values for hematological, coagulation, clinical chemistry and urinalysis parameters reported for the treated groups that exhibited statistically significant changes from controls are also within the range of normal for SD rats as reported by Derelanko [18], lending support to the study investigator’s conclusion that the changes observed in treated animals are not adverse. No abnormal gross findings were observed at necropsy, and all histopathological lesions that were observed were considered to be incidental or spontaneous due to similar incidences in control and high dose animals. A few changes in organ weights were observed in treated animals compared to controls but they were not considered to be adverse due to lack of dose–response relationship or corresponding histopathological or clinical chemistry findings.

*W. cibaria* CMU was not mutagenic in an OECD Guideline 471 bacterial reverse mutation assay which tested concentrations up to the limit concentration of 5000 µg/plate (in the presence and absence of metabolic activation) or clastogenic in an OECD Guideline 473 in vitro chromosome aberration assay in cultured CHO-k1 cells. Both of the assays met the criteria for validity that are required by the OECD [14, 15]. The results of an OECD 474 Guideline micronucleus study in the mouse show that *W. cibaria* CMU is not clastogenic or aneugenic up to 5000 mg/kg bw. Toxicity was not noted in the micronucleus study—there was no effect of the test substance on body weight or PCE/PCE + NCE ratio and no abnormal clinical signs were noted. However, the study is valid and fit for purpose because the assay was sensitive enough to detect micronucleated PCE (as indicated by the positive control response), and the highest dose of *W. cibaria* CMU administered was the same dose evaluated for safety in rats. Further, the 5000 mg/kg bw dose is higher than the limit dose of 2000 mg/kg bw recommended by the OECD [16].

In conclusion, the results of the studies described in this manuscript show that oral administration of up to 5000 mg/kg bw/day (1.8 × 10^9^ CFU/kg bw/day) *W. cibaria* CMU is safe in rats and suggest that the strain could be safely consumed by humans. Furthermore, *W. cibaria* CMU is non-genotoxic as shown by the negative results of in vitro bacterial reverse mutation and CHO-k1 cell chromosome aberration studies and the in vivo micronucleus study in mice. This study is the first study examining the potential of a *W. cibaria* strain to cause genetic toxicity and subchronic toxicity in rats according to OECD guidelines.

## Data Availability

The data presented in this study are available on request from the corresponding author.
